# Whole-Transcriptome Sequencing: A Powerful Tool for Vascular Tissue Engineering and Endothelial Mechanobiology

**DOI:** 10.3390/ht7010005

**Published:** 2018-02-21

**Authors:** Anton G. Kutikhin, Maxim Yu. Sinitsky, Arseniy E. Yuzhalin, Elena A. Velikanova

**Affiliations:** 1 Research Institute for Complex Issues of Cardiovascular Diseases, 6 Sosnovy Boulevard, Kemerovo 650002, Russia; sinimu@kemcardio.ru (M.Y.S.); veliea@kemcardio.ru (E.A.V.); 2 Department of Oncology, Cancer Research UK and Medical Research Council Oxford Institute for Radiation Oncology, University of Oxford, Old Road Campus Research Building, Roosevelt Drive, Oxford OX3 7DQ, UK; arseniy.yuzhalin@oncology.ox.ac.uk

**Keywords:** high-throughput techniques, whole-transcriptome sequencing, RNA sequencing, cardiovascular diseases, endothelial mechanobiology, vascular tissue engineering, shear stress, cyclic strain, endothelial cells, endothelial progenitor cells

## Abstract

Among applicable high-throughput techniques in cardiovascular biology, whole-transcriptome sequencing is of particular use. By utilizing RNA that is isolated from virtually all cells and tissues, the entire transcriptome can be evaluated. In comparison with other high-throughput approaches, RNA sequencing is characterized by a relatively low-cost and large data output, which permits a comprehensive analysis of spatiotemporal variation in the gene expression profile. Both shear stress and cyclic strain exert hemodynamic force upon the arterial endothelium and are considered to be crucial determinants of endothelial physiology. Laminar blood flow results in a high shear stress that promotes atheroresistant endothelial phenotype, while a turbulent, oscillatory flow yields a pathologically low shear stress that disturbs endothelial homeostasis, making respective arterial segments prone to atherosclerosis. Severe atherosclerosis significantly impairs blood supply to the organs and frequently requires bypass surgery or an arterial replacement surgery that requires tissue-engineered vascular grafts. To provide insight into patterns of gene expression in endothelial cells in native or bioartificial arteries under different biomechanical conditions, this article discusses applications of whole-transcriptome sequencing in endothelial mechanobiology and vascular tissue engineering.

## 1. Background

Over the last decades, cardiovascular diseases have remained a leading cause of death worldwide, however, there has been a notable decrease in both age-standardized death rates and age-standardized, disability-adjusted life years per 100,000 population [1,2]. The abovementioned decline is largely due to progress in drug discovery and development, improvements in interventional cardiology and cardiovascular surgery, and the widespread implementation of evidence-based medicine that increases the availability and efficacy of cardiovascular care. In line with clinical advances, there is an abundance of ongoing basic and translational research intended to develop novel, versatile tools for cardiovascular medicine. This involves a number of scientific fields, such as molecular and cell biology, tissue engineering, personalized medicine, and nanomedicine.

The advent of high-throughput techniques (e.g., next-generation sequencing, proteomic arrays, and high-resolution mass spectrometry) has revolutionized biomedical research. It is now possible to uncover the multiple signaling pathways responsible for both physiological and pathological processes, and to pinpoint the molecular culprits of human disease. Such an approach has been actively employed in cancer research [3,4,5], atherosclerosis [6,7,8], chronic obstructive pulmonary disease [9], chronic kidney disease [10,11,12,13], endocrine disorders [14,15,16], autoimmune disorders [17,18], and neurodegenerative disorders [19,20,21]. Moreover, the integration of these data has led to the development of network biology and high-throughput drug screening [22,23,24,25,26,27,28,29,30]. Applying high-throughput methods frequently requires a collaboration between specialists from different fields, including omics, bioinformatics, life sciences, health sciences, and engineering.

Current cardiovascular surgery requires a small-diameter (≤6 mm) vascular graft for both arterial replacement and bypass surgery, yet this clinical need is still unmet [31,32,33,34,35]. Unfortunately, biostable synthetic vascular grafts have demonstrated poor results, reporting patency rates of 40% at six months and only 25% at three years postimplantation [32]. This is due to thrombosis and intimal hyperplasia secondary to a lack of endothelialization, low blood flow, and compliance mismatch [33,35]. Other reasons include infection, calcification, and the formation of pseudoaneurysms [31,34]. In addition, such grafts lack the capacity for adaptive growth and often result in a repeated surgery and unacceptable long-term outcomes [31,32]. This is of particular concern for pediatric patients with congenital heart disease, who frequently undergo vascular graft surgery at a young age [32].

Vascular tissue engineering has emerged as a promising approach for producing mechanically competent and biocompatible small-diameter vascular substitutes [31]. Most research in this field deploys polymer tubular scaffolds to provide a surface for cell attachment, proliferation, and migration, resulting in the formation of new vascular tissue and followed by the degradation of the scaffold [36]. Clinical demands for ready-to-use, biodegradable, small-diameter vascular grafts are present in all fields of cardiovascular surgery; e.g., heart surgery (treatment of coronary artery disease), vascular surgery (distal revascularization of lower limbs), neurosurgery (repair of intracranial arteries), pediatric cardiovascular surgery (treatment of congenital heart disease), and microsurgical reconstruction after severe hand traumas [31].

To minimize the risk of thrombosis and inflammation, vascular grafts should be covered by a monolayer of autologous endothelial cells (ECs); this also meets the concept of personalized medicine, which has been rapidly developing during the last decade [37,38,39,40,41,42,43,44]. As mature ECs have a relatively low proliferation rate [45], endothelial progenitor cells (EPCs) are actively used to induce endothelialization of the tubular scaffolds in vitro [46,47,48]. Autologous EPCs can be obtained by differentiation of peripheral blood-derived mononuclear cells cultured on fibronectin-coated dishes in endothelial basal medium supplemented with fetal calf serum, epidermal growth factor, vascular endothelial growth factor 165, basic fibroblast growth factor, insulin-like growth factor 1, hydrocortisone, ascorbic acid, and heparin [49,50,51,52], or by direct isolation from the blood utilizing magnetic-activated cell sorting [53,54,55].

According to research, shear stress preconditioning promotes endothelialization [51,56,57,58,59,60,61,62] and further differentiation of EPCs into mature ECs [51,57,58,62,63,64,65,66,67,68,69,70,71,72,73,74] that contain both anti-thrombotic and anti-atherosclerotic phenotypes [58,65,72,73,75,76,77,78,79,80,81]. Endothelial alignment is critical for both EPCs and mature ECs to maintain vascular homeostasis in response to shear stress [51,56,57,58,59,60,61,62,82,83,84,85,86,87,88,89]. Unidirectional laminar flow, characterized by high shear stress and the synchronous performance of shear stress and cyclic strain, induces EC elongation and alignment in the flow direction, with the subsequent generation of tight junctions between ECs and the eventual formation of a confluent EC monolayer [51,56,57,58,59,60,61,62,82,83,84,85,86,87,88,89,90,91]. Further, laminar flow governs cell turnover and vascular tone, which regulate vascular permeability and sustain an anti-inflammatory microenvironment [51,64,72,82,83,84,85,86,92]. Mechanistically, the atheroprotective effect of unidirectional laminar flow was recently found to be largely determined by the decreased activity of Hippo pathway members Yes-associated protein (YAP) and transcriptional coactivator with PDZ-binding motif (TAZ) in ECs that suppress c-Jun N-terminal kinase (JNK) signaling and downregulate the expression of pro-inflammatory genes [93,94,95]. In contrast, turbulent, multidirectional, oscillatory flows are notable for their pathological low shear stress, asynchronous shear stress action, and cyclic strain on the vascular wall [82,83,84,85,86,90,91]. They disrupt EC alignment, result in the acquisition of round or polymorphic shapes by ECs, accelerate their apoptosis, impair the regulation of vascular tone and permeability, and stimulate a pro-inflammatory microenvironment [82,83,84,85,86]. Disturbed flow was shown to elicit a proatherogenic response through upregulation of the pro-inflammatory transcription factor nuclear factor κβ (NF-κβ) [91,96] and an inhibition of the atheroprotective factor Kruppel-like factor 2 (KLF2) [96] and the antioxidant factor nuclear factor (erythroid-derived 2)-like 2 (Nrf2) at the DNA binding step [96,97].

However, studies on the mechanobiology of EPCs are mostly limited to classical molecular biology techniques and therefore explore only a minor percentage of relevant biochemical pathways. Furthermore, cell signaling networks are characterized by a considerable redundancy in master regulator molecules that exert pleiotropic effects on different molecular pathways. The combination of high-throughput methods (e.g., whole-transcriptome sequencing, or protein arrays such as dot blot) and routine molecular biology techniques (e.g., quantitative polymerase chain reaction (qPCR) or conventional Western blotting) is capable of determining the activity of numerous biochemical pathways simultaneously. Although expensive, this approach provides a large amount of data drawn from high-throughput techniques, which are then validated through the use of classical methods. In addition, one can highlight a synergistic combination of whole-transcriptome sequencing and laser capture microdissection, which is a technique designed for the isolation of specific cells or tissues at a microscopic (even at a single-cell) resolution that enables high-throughput analysis of the genomic data from a source/region of interest. In this review, we focus on whole-transcriptome sequencing, as it combines transparent and efficient data processing with large data output, relatively low costs, and simple verification by qPCR.

Currently, there are two main approaches to the analysis of the whole transcriptome: whole-transcriptome shotgun sequencing (RNA sequencing, RNA-seq), and whole-transcriptome, target/tag sequencing, with or without restriction digestion [98,99,100]. The former enables the characterization of both mRNA and non-coding RNA, regardless of polyadenylation, while the latter exclusively targets mRNA, i.e., all polyadenylated transcripts within the transcriptome [98,99,100]. To improve the identification of low-abundance transcripts, an increase in the depth of the sequencing can be accomplished by removing irrelevant RNAs. The enrichment of the relevant part of the transcriptome prior to sequencing is achieved by rRNA depletion (e.g., utilizing biotin–streptavidin-based bead systems) or the selection of poly-A tailed transcripts from total RNA (e.g., using the Oligo dT magnetic bead system) when employing whole-transcriptome shotgun sequencing or whole-transcriptome target/tag sequencing, respectively. Generally, whole-transcriptome shotgun sequencing is characterized by a higher output but is more expensive compared to whole-transcriptome target/tag sequencing. Therefore, the choice between these two techniques is unique to each particular experiment. We further present demonstrative examples of how whole-transcriptome sequencing can be used in endothelial mechanobiology and vascular tissue engineering studies.

## 2. Application of Whole-Transcriptome Sequencing to Endothelial Mechanobiology Studies

Whole-transcriptome sequencing has been widely utilized to investigate the response of various EC cultures to different types of shear stress, of variable intensity and at sequential time points, as well as to the addition of bioactive molecules. A number of milestone papers employed this approach to discover specific patterns of gene expression under the abovementioned conditions. The genome-wide expression profiling of human umbilical vein ECs (HUVECs) exposed to unidirectional laminar shear stress (from 24 h up to six days of culture) identified three clusters of gene response: (1) downregulated by the shear stress but unaffected by the tumor necrosis factor (TNF)-α; (2) upregulated by the TNF-α treatment both under static and shear conditions; and (3) upregulated by the shear stress but downregulated or unchanged after the TNF-α exposure [101].

In HUVECs cultured under prolonged (six days) pulsatile flow in comparison with static conditions, the gene expression of a potent vasoconstrictor endothelin-1 (EDN1) was downregulated while that of the pro-thrombotic molecule plasminogen activator inhibitor 1 (PAI1) remained unchanged [101]. In contrast, the expression of *KLF2*, *NOS3*, and *THBD* genes encoding KLF2, endothelial nitric oxide synthase, and thrombomodulin—all of which are anti-thrombotic or anti-atherosclerotic molecules—was significantly higher in sheared than in statically cultured ECs [101]. In addition, exposure to the shear stress also suppressed the expression of pro-inflammatory *SELE*, *VCAM1*, *CCL2*, *CX3CL1*, *CXCL6*, and *CXCL8* genes encoding E-selectin, vascular cell adhesion molecule 1, C-C motif ligand 2 (monocyte chemoattractant protein 1), C-X3-C motif ligand 1 (fractalkine), C-X-C motif ligand 6 (granulocyte chemotactic protein 2), and interleukin-8, respectively [101,102]. Such a pattern of gene expression regulated by shear stress was induced through the KLF2-mediated inhibition of the nuclear activity of activating transcription factor 2 (ATF2), the regulation of SMAD/activator protein-1 (AP-1) axis, and the involvement of the Nrf2 that binds the antioxidant response element (ARE) [101]. A follow-up study by the same research group confirmed the upregulation of both KLF2- and Nrf2-induced transcriptomes by shear stress [103]. Highlighting the importance of KLF2 in maintaining endothelial homeostasis, its molecular inducers such as statins [104], resveratrol [105], suberanilohydroxamic acid [106] and tannic acid [107] were recently suggested as a potential therapy for atherosclerosis treatment and prophylaxis.

Another study that employed genome-wide expression profiling identified 32 genes up- or downregulated in HUVECs upon 24 h of exposure to high shear stress compared to low shear stress [108]. Genes for C-X-C chemokine receptor type 4 (CXCR4), caspase recruitment domain-8 (CARD8) and apoptosis-associated protein 2 (THPA2)—which are mediators of inflammation and apoptosis—were under-expressed at high shear in comparison with low shear stress, however, the reverse effect was observed for tumor necrosis factor α-induced protein 3 (TNFAIP3), which is an inhibitor of the cytokine-induced activation of NF-kB in ECs [108]. The gene expression of the acyl-CoA synthetase family member 3 (ACSL3), which activates long-chain fatty acids and therefore enhances the synthesis of cellular lipids, was lowered at high shear stress, in keeping with its atheroprotective role [108]. As defined by RNA sequencing, the exposure of HUVEC cultures to a high laminar shear stress for 72 h also decreased the expression of genes responsible for glycolysis and intimal acidification, including those encoding hexokinase (HK1/2), phosphofructokinase (PFK1), and 6-phosphofructo-2-kinase/fructose-2,6-biphosphatase (PFKFB3) in a KLF2-dependent manner [109].

Further, whole-transcriptome sequencing can be applied to study the impact of gene knockdown (e.g., by means of small interfering RNA (siRNA) or short hairpin RNA (shRNA)) or gene overexpression (e.g., using adenoviral or lentiviral transfection) on the global gene expression in ECs. For instance, Maleszewska et al. showed that the knockdown of *EZH2*, a major methyltransferase in the Polycomb repressive complex-2, which methylates histone H3 at lysine-27 and therefore controls gene expression [110], increased the expression of 2042 genes, particularly those responsible for cell adhesion, and reduced the expression of 2654 genes, primarily those governing the cell cycle (e.g., *CCNA1*, *CCNB1*, and *CCNB2*) in HUVECs exposed to the high shear stress for 72 h [111]. Similarly, *KLF4* gene overexpression enhanced biosynthesis of the nitric oxide and upregulated cholesterol efflux and oxidation while downregulating cholesterol synthesis and mitigating inflammation in HUVECs, largely via transactivation of cholesterol-25-hydroxylase, an enzyme converting cholesterol to 25-hydroxycholesterol, and liver X receptor, a transcription factor regulating cholesterol synthesis and metabolism [112].

As human ECs isolated from distinct vascular regions are characterized by significant diversity, and both atherosclerosis and vascular grafting involve arteries, arterial ECs are preferable to HUVECs as a model for studying endothelial mechanobiology [113]. Compared to primary human coronary artery ECs (HCAECs) cultured under high unidirectional laminar shear stress for 24 h, 8177 (50%) and 9369 (57%) of genes were differentially expressed in HCAECs cultured for the same time under either bidirectional oscillatory shear stress or static conditions, respectively [114]. However, only 1618 (10%) of genes were differentially expressed in cells cultured under bidirectional oscillatory shear stress and under static conditions [114]. For cells cultured under any of the three abovementioned conditions, the fold change in the expression level obtained using qPCR was similar to that obtained using RNA sequencing for the following core shear-sensitive genes: *KLF2*, angiopoietin-2 (*ANGPT2*), *NOS3*, *VCAM1*, *CXCR4*, inhibitor of DNA binding 1 (*ID1*), fatty acid binding protein 4 (*FABP4*), hyaluronoglucosaminidase 2 (*HYAL2*), *KLF11*, serpin peptidase inhibitor member 2 (*SERPINE2*), LIM domain 7 (*LMO7*), chemokine (C-C motif) ligand 14 (*CCL14*), TEK tyrosine kinase (*TEK*), latexin (*LXN*), chromosome 10 open reading frame 10 (*C10orf10*), and ephrin A1 (*EFNA1*) [114]. The majority of genes that were differentially expressed under static conditions versus laminar shear stress (77%) and laminar versus oscillatory shear stress (88%) were overlapped and indicative of the existence of similar gene expression patterns in cells cultured under static conditions and at oscillatory shear stress; this was also confirmed by qPCR [114].

In addition to arterial and vein ECs, whole-transcriptome sequencing has been used for the investigation of shear stress effects on the global gene expression of endocardial ECs (EECs). An analysis of distinct regions within the porcine left ventricle showed that the number of differentially expressed genes in apex-mid-ventricle, base-mid-ventricle, and base-apex comparisons was 0, 325, and 1051, respectively. These differences in gene expression patterns were consistent with those in shear stress values between the regions [115,116]. Notable discrepancies between base and apex were detected regarding the increased expression of *TFPI* (tissue factor pathway inhibitor) and *PTGIS* (prostacyclin synthase) genes, which encode two major anticoagulant proteins, in the apex EECs intrinsically exposed to low shear stress [115,116]. Furthermore, *HS6ST2* and *NRP1* genes encoding the components of glycocalyx, which provides electrical repulsion of activated platelets [117], were upregulated in the apex compared to the base [115,116]. A pathway analysis found apical upregulation of the genes responsible for translation initiation and oxidative phosphorylation [115,116]. This can reflect a physiological response of the apex EECs to avoid excessive coagulation and thrombosis.

Another important issue is the temporal variation in the gene expression profile in ECs under shear stress. Time-resolved RNA sequencing of HUVECs exposed to laminar or oscillatory shear conditions revealed a significant number of differentially expressed genes at ten time points during 24 h [118]. The expression of genes governing cell cycle (e.g., those responsible for the G1/S transition) commenced to differ between four and six hours after the initial exposure to shear, being substantially higher at the oscillatory flow [118]. In keeping with these findings, a notable upregulation of gene pathways responsible for ribosomal production and activity as well as for global protein expression and degradation was observed at later time points [118]. Genes related to the production of reactive oxygen species were overexpressed as early as hour two, while those encoding certain antioxidative enzymes, such as catalase, glucose-6-phosphate dehydrogenase, or NAD(P)H dehydrogenase (quinone) 1 were downregulated at hour nine to hour twelve [118]. However, genes for mitochondrial superoxide dismutase and metallothioneins were overexpressed at hours four and nine, respectively, while those for peroxiredoxins and glutathione peroxidases were upregulated from hour twenty [118]. Consistent with the current understanding of mechanotransduction and atherosclerosis [82,83,84,85,86], the expression of the pro-inflammatory genes (e.g., *NFKB1*, *VCAM1*, *SELE*, *CCL2*, and *CXCL8*) was significantly increased in the ECs between hours two and six [118]. Oscillatory shear stress was associated with the endothelial-to-mesenchymal transition expression pattern (downregulation of endothelial marker genes *CD34*, von Willebrand factor (*VWF*), and *NOS3* from hour six, along with the upregulation of mesenchymal marker genes cadherin-2 (*CDH2*), fibulin-5 (*FBLN5*), and tropomyosin α-1 chain (*TPM1*) from hour twelve) [118]. Expression of the *HIF1A* gene-encoding, hypoxia-inducible factor was elevated from hour four [118], in concert with the crucial role of hypoxia in the development of atherosclerosis [119]. The application of whole-transcriptome sequencing in endothelial mechanobiology has not been limited to the identification of mechanosensitive genes, but was also extended to the determination of the flow-regulated, long, non-coding RNAs (lncRNAs) constituting a large part of the transcriptome [105]. For instance, LINC00341, an abundant lncRNA that has a sequence verified by qPCR and is markedly expressed under pulsatile shear conditions, has been shown to suppress TNF-α-induced vascular cell adhesion molecule (VCAM)-1 expression and monocyte adhesion [120].

Regarding translational medicine, whole-transcriptome sequencing has proved useful in the implementation of patient-oriented approaches in cardiovascular medicine, i.e., for the transfer of the patient’s data to the bench to uncover a molecular basis for corresponding diseases. By using 3D-computational tomography angiography (3D-CTA) and by calculating the shear stress in three patients with unruptured intracranial aneurysms and different arterial geometries, Aoki et al. obtained shear stress values and flow type (low shear stress at either laminar or turbulent flow and high shear stress at the laminar flow) in primary cultures of human carotid artery ECs (HCtAECs) [102]. Subsequent RNA sequencing, verified by qPCR, identified that the transcriptomic signatures associated with nucleosome assembly, mitotic cell cycle, DNA replication, and the recruiting/adhesion of immune cells tend to be overrepresented in cells cultured at low shear stress, in particular under turbulent flow conditions, when compared to those cultured at high shear stress [102]. This corresponds to seminal findings that demonstrate that the accelerated turnover of ECs [121,122] and considerable inflammation [123] occur in regions of turbulent flow.

Cyclic strain, also called cyclic stretch, is another major hemodynamic force affecting the differentiation of EPCs and the gene expression profile of mature ECs in the human body [124,125,126,127,128]. The genome-wide miRNA microarray analysis of HUVECs subjected to the cyclic strain for 24 h (60 cycles/minute) detected 20 upregulated and 18 downregulated miRNAs when compared to statically cultured cells [129]. These miRNAs were involved in numerous signaling pathways, including those regulating apoptosis, cell cycle control, differentiation, and inflammation [129]. Similar results were obtained in the genome-wide analysis of miRNAs in HUVECs exposed to the cyclic strain for three hours (60 cycles/minute) [130].

## 3. Application of Whole-Transcriptome Sequencing to Vascular Tissue Engineering Studies

Unfortunately, there have been few attempts to utilize whole-transcriptome sequencing to evaluate the response of ECs on their attachment to tubular grafts. However, even biocompatible polymers, such as extracellular matrix proteins, showed considerable variation in their ability to enhance EPC adhesion and further differentiation under shear stress conditions [69,131]. Compared to type I collagen and laminin, fibronectin better promotes the differentiation of EPCs, possibly because of its profound stimulative effects on the shear stress-related expression of integrins [69,131]. Integrin-mediated signaling is required to activate the paxillin/focal adhesion kinase (FAK)/rat sarcoma (RAS)/extracellular signal-regulated kinase (ERK) pathway and induce endothelial lineage differentiation [69,131].

The micro- and nanopatterning of polymer surfaces has been suggested as a promising approach that increases biocompatibility and therefore improves cell attachment [132,133]. A genome-wide expression analysis found that 3303 genes were significantly underexpressed, while 905 genes were substantially overexpressed in primary human dermal microvascular ECs (HDMECs) cultured on micropatterned polydimethylsiloxane (PDMS) substrates, when compared to their non-patterned counterparts [134]. In particular, a notable upregulation of RNA processing, DNA replication, and DNA repair pathways in HDMECs on the micropatterned substrates was noted, indicating their accelerated proliferation [134]. Genes responsible for extracellular matrix degradation and vascular remodeling (e.g., those encoding matrix metalloproteinase 1 (MMP1) and 16 (MMP16)) were also overexpressed on micropatterned surfaces [134]. Among the downregulated pathways were transmembrane receptor activity, G-protein-coupled receptor activity, and ion channel activity [134].

In 2006, Takahashi and Yamanaka demonstrated that four transcription factors (c-Myc, Oct4, Sox2, and Klf4) are responsible for transforming adult fibroblasts into induced pluripotent stem cells (iPSCs) that exhibit a similar morphology, proliferation pattern, gene expression profile, and surface markers to embryonic stem cells [135,136]. The iPSCs showed an almost inexhaustible capacity for replication and were able to differentiate into cell types of all three germ layers [135,136]. Subsequent studies confirmed these findings, paving the way for the investigation of iPSC applications in various biomedical fields [137,138,139]. As the use of iPSCs does not involve ethical considerations, these cells have been suggested as a promising tool for tissue engineering and regenerative medicine. For instance, iPSCs can be differentiated into ECs [140], vascular smooth muscle cells [140], and cardiomyocytes [140,141]. This feature of iPSCs was successfully applied in the generation of autologous ECs [142] and smooth muscle cells [142,143,144] to seed tissue-engineered vascular grafts. Recent studies revealed that immature iPSC-ECs can be differentiated into mature arterial ECs in as early as 24 h using a biomimetic flow bioreactor [145] and will acquire a physiological phenotype under shear stress conditions [146,147]. As iPSC-derived ECs adequately respond to growth factors, inflammatory and thrombotic stimuli, and hemodynamic forces, they were proposed as a perfect functional lining for bioartificial blood vessels [148,149]. Furthermore, iPSC-ECs cultured along with fibroblasts form 2D and 3D capillary networks are potentially capable of facilitating perfusion of non-vascular, tissue-engineered constructs [148,149,150,151,152]. One can apply such 3D models to the study of endothelial mechanobiology under pulsatile flow conditions and mimic the anatomy of different vessels, resulting in a simulation where laminar or turbulent flow pattern occurs. This can be realized utilizing microfluidic devices for microvascular research, or conventional bioreactors to imitate the hemodynamics of small to large arteries. The latter approach often requires the use of polymer scaffolds. Whole-transcriptome sequencing can additionally improve such experimentation, permitting a demarcation of endothelial differentiation stages with genome-wide coverage and simultaneous detection of molecular responses across multiple pathways at sequential time points.

## 4. Conclusions and Future Directions

**Figure 1:**
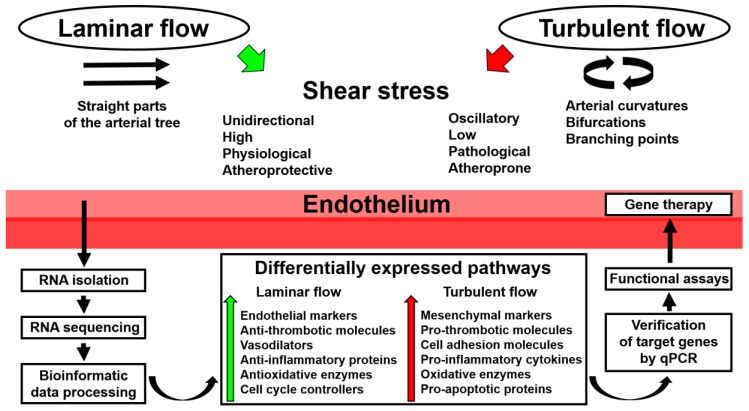
Application of whole-transcriptome sequencing in endothelial mechanobiology. qPCR: quantitative polymerase chain reaction.

In recent years, a number of studies have demonstrated the advantage of utilizing whole-transcriptome sequencing in endothelial mechanobiology and vascular tissue engineering. Such an approach can be successfully applied to any cell line or tissue regardless of experimental conditions, with the RNA yield and quality of the extraction sample being the only limiting factors. Moreover, RNA amplification is capable of maximizing the amount of RNA needed for subsequent sequencing or reverse transcription. As discussed above, whole-transcriptome sequencing can be employed for: 1) routine global gene expression profiling of ECs cultured with various biomechanical cues; 2) deep investigation of endothelial responses to physiological changes, such as the addition of bioactive factors; 3) deciphering spatiotemporal variation in the expression of multiple signaling pathways; 4) testing patient-specific biophysical conditions on EC cultures; and 5) determining transcriptomic signatures in ECs during their culture on distinct polymer surfaces. A scheme illustrating the use of whole-transcriptome sequencing in studying endothelial response to shear stress is depicted in Figure 1.

It is also important to note that the microarray/RNA-seq data generated in different studies are deposited in publicly available databases. This allows researchers to mine gene profiling data in order to perform various *in silico* analyses of the transcriptome. Examples of basic bioinformatics analyses of such published datasets were recently described by Xu [153].

There are still gaps in our knowledge of the gene expression patterns in certain arterial cells in response to shear stress, cyclic strain, or both combined. Another level of complexity is the analysis of the mentioned expression profiles in different segments of the arterial tree, which often require laser capture microdissection in order to isolate regions of interest. The proper engineering of biodegradable vascular grafts must include tracing global gene expression in the newly formed tissue. In addition, transcriptomic profiling can significantly extend our understanding of EPC maturation once they are attached to distinct polymers (e.g., extracellular matrix proteins). To improve upon the respective experiments, whole-transcriptome sequencing must be combined with qPCR and protein expression should be measured using routine molecular biology methods or proteomics techniques.

With the growing amount of cardiovascular research using whole-transcriptome sequencing, researchers are now able to perform an in silico analysis of gene expression responses to various biophysical stimuli in ECs from different sources. Such an approach can be useful when comparing site-specific physiological features and can provide further insight into the origin of vascular diseases. To summarize, whole-transcriptome sequencing is currently recognized as a versatile tool in physiological genomics and can be broadly implemented in various branches of vascular biology.
